# Strain variation in *Candida albicans* glycolytic gene regulation

**DOI:** 10.1128/msphere.00579-24

**Published:** 2024-10-21

**Authors:** Min-Ju Kim, Amelia M. White, Aaron P. Mitchell

**Affiliations:** 1 Department of Microbiology, University of Georgia, Athens, Georgia, USA; Virginia-Maryland College of Veterinary Medicine, Blacksburg, Virginia, USA

**Keywords:** *Candida albicans*, strain variation, glycolysis, gene regulation

## Abstract

Central carbon metabolism is vital for the proliferation of *Candida albicans*, a fungus that is prominent as a commensal and pathogen. Glycolytic genes are activated by overlapping activities of the transcription factors Tye7 and Gal4, as shown by studies in the SC5314 genetic background. However, regulatory relationships can vary among *C. albicans* isolates. Here, we analyzed Tye7- and Gal4-related phenotypes in five diverse clinical isolates of *C. albicans*. We tested growth properties and gene expression impact through Nanostring profiling and, for the two strains SC5314 and P87, RNA sequencing. Our results lead to three main conclusions. First, the functional redundancy of Tye7 and Gal4 for glycolytic gene activation is preserved among all strains tested. Second, at the gene expression level, strain P87 is an outlier with regard to *tye7*Δ/Δ impact, and strain SC5314 is an outlier with regard to *gal4*Δ/Δ impact. Third, while Gal4 is well known to be dispensable for induction of the *GAL1*, *GAL7*, and *GAL10* galactose-specific metabolic genes, we find that *gal4*Δ/Δ mutants of several strains have a mild galactose fermentation defect, as assayed by growth on galactose with the respiration inhibitor antimycin A. Our findings indicate that even a central metabolic regulatory network is subject to strain variation and illustrates an unexpected genotype-phenotype relationship.

The fungal commensal and pathogen *Candida albicans* rely upon metabolic flexibility to colonize and infect host niches. Central carbon metabolism is governed by two regulators, Tye7 and Gal4, as defined in the reference strain SC5314. Here, we have explored the impact of Tye7 and Gal4 on carbon utilization and gene expression across five diverse *C. albicans* clinical isolates. Novel aspects of this study are the finding that even a central metabolic regulatory network is subject to strain variation and the observation of an unexpected mutant phenotype.

## INTRODUCTION

The fungus *Candida albicans* can interact with human hosts as both a commensal and a pathogen (1). It is capable of infecting almost any tissue. The mortality rate for deep tissue infection is over 60% (2), a reflection of the limited antifungal arsenal (3). Metabolic flexibility plays a central role in the ability of *C. albicans* to infect and persist in diverse body sites, and for this reason, metabolic regulation may be considered a virulence determinant (4).

Among the major regulators of carbon metabolism in *C. albicans* are the transcription factors (TFs) Tye7 and Gal4 (5). Both Tye7 and Gal4 bind glycolytic gene promoters, and function redundantly to activate expression of the core genes of glycolysis (5), those required to metabolize glucose to pyruvate. Their impact on hexose utilization is most evident when *C. albicans* is forced to grow through fermentation (5), which has a much lower yield of ATP per hexose molecule than respiration.

Tye7 is a basic helix-loop-helix transcription factor that is orthologous to the accessory glycolytic regulator Tye7/Sgc1 of *Saccharomyces cerevisiae* (6). Its binding motif, 5′-CANNTG-3′, is enriched in glycolytic gene promoters (5). The key role of Tye7 in glycolytic gene activation impacts *C. albicans* pathogenicity traits that include virulence (5), biofilm formation (7), and hypoxic responses (5, 8). Tye7 binds to 271 targets in the rich glucose medium yeast extract-peptone-dextrose (YPD), whereas Gal4 binds to only 98 targets under those conditions (5). Hence, Tye7 regulates many genes independently of Gal4, including those involved in the metabolism of glycogen, trehalose, and glycerol (5). Tye7 is considered a more central regulator of carbohydrate metabolism than Gal4 (5).

Gal4 is a zinc cluster [Zn(II)_2_Cys_6_] transcription factor that is orthologous to *S. cerevisiae* Gal4, the well-known *GAL1-GAL7-GAL10* galactose gene activator (9). In *C. albicans* and other CTG clade yeasts, Gal4 has lost its role in *GAL* gene activation and acquired a role in glycolytic gene activation (10). The Gal4 binding motif, 5′-CGG(N_11_)CCG-3′, is enriched in glycolytic gene promoters (5). Askew and colleagues found that Gal4 expression and promoter-binding activity are higher during glucose growth than during glycerol growth (5), leading them to propose that activation of glycolytic genes by Gal4 assists Tye7 to increase glycolytic flux during growth on fermentable carbon sources (5).

Most mechanistic studies of *C. albicans*, including the characterization of Tye7 and Gal4, have used strain SC5314 and its derivatives. Natural variation among *C. albicans* isolates is extensive, though, as indicated by both genome sequences (11–13) and phenotypic diversity (12–20; reviewed in reference 21). One result of strain variation is that a defined mutation, such as an engineered deletion allele, can cause different phenotypes in different strains (22–25). Sometimes the differences are quantitative, affecting the magnitude of a mutant defect. Sometimes the differences seem more qualitative, affecting whether a mutant defect is observed at all. Variation in genotype-phenotype relationships is found in essentially all organisms, so its existence in *C. albicans* is not surprising (22–25). However, exclusive use of a single strain or genetic background can skew our understanding of gene function. In addition, the extent of phenotypic consistency among strains can help distill a group of genes into the most promising therapeutic targets.

We chose to analyze Tye7 and Gal4 specifically because of their roles in the regulation of central carbon metabolism (5, 10). Regulators of a critically important process may be under strong selection to maintain their activities. On the other hand, the overlap among their targets may enable functional divergence despite strong selection. Here, we have initiated an analysis of Tye7- and Gal4-related phenotypes and gene expression features in five diverse clinical isolates of *C. albicans*.

## MATERIALS AND METHODS

### Strains and media

*C. albicans* strains SC5314, P76067, P57055, P87, and P75010 and their derived *his1*∆/∆ mutants (15) were used as transformation recipients. Fungal strains were grown overnight at 30°C in YPD liquid medium (2% Bacto peptone, 2% dextrose, and 1% yeast extract) in a tissue culture rotator. *C. albicans* transformants were selected on CSM-HIS (0.67% yeast nitrogen base without amino acids, 0.079% CSM-HIS, and 2% dextrose) for His + isolates or YPD plus NAT (2% Bacto peptone, 2% dextrose, 1% yeast extract, and 400 mg/mL for nourseothricin-resistant [NatR] isolates). All strains were stored as glycerol stocks at −80°C. Strains used in this study are listed in Table S1.

### Phenotypic assays

For phenotypic assays, strains were grown either on YPD plates or CSM (0.67% yeast nitrogen base without amino acids, 0.074% CSM) plates with carbon sources at 0.2% concentration as indicated (glucose, fructose, and galactose). Where indicated, media included 2 µg/mL antimycin A (Sigma-Aldrich, St. Louis, MO).

### Oligos and DNA

Oligos and plasmids used in this study are listed in Table S1. Gene sequences were retrieved from the Candida Genome Database (26) or FungiDB (27).

### Single deletion strain construction

Construction of the *tye7*∆/∆ and *gal4*∆/∆ in clinical isolates was employed as described previously. Briefly, there were three steps. (i) Construction of single guide RNA (sgRNA) expression cassette. The SNR52 promoter was amplified with primers SNR52/F and SNR52/R_TYE7 (SNR52/R_GAL4) from plasmid pV1093 (28); the sgRNA scaffold was amplified with primers sgRNA/F_TYE7 (sgRNA/F_GAL4) and sgRNA/R from plasmid pV1093 (28). Then fusion PCR with nested primers SNR52/N and sgRNA/N was used to amplify the final sgRNA expression cassette. (ii) Construction of *tye7*∆*::r1HIS1r1* (*gal4*∆*::r1HIS1r1*) cassette. One of the two halves of the *tye7*∆*::r1HIS1r1* (*gal4*∆*::r1HIS1r1*) cassette was amplified from pMH01 using primers His1 CRIME/F and TYE7 Del rHISrKpnI/R (GAL4 Del rHISrKpnI/R); the other half of the *tye7*∆*::r1HIS1r1* (*gal4*∆*::r1HIS1r1*) cassette was amplified from pMH02 using primers His1 CRIME/R and TYE7 Del rHISrSapI/F (GAL4 Del rHISrSapI/F) (29). (iii) The Cas9 cassette was amplified from the plasmid pV1093 by primers CaCas9/F and CaCas9/R (28). The Cas9 cassette was transformed with *TYE7* sgRNA (*GAL4* sgRNA) cassette and *tye7*∆*::r1HIS1r1* (*gal4*∆*::r1HIS1r1*) cassette into five His− isolates via the transient CRISPR-Cas9 system as described in Min et al. (30). Transformants were verified by primers TYE7 Check/F (GAL4 Check/F) and CdHIS1 Check int/R; primers TYE7 Check/F (GAL4 Check/F) and TYE7 Check int/R (GAL4 Check int/R).

### Double deletion strain construction

Construction of the *gal4*∆/∆ *tye7*∆/∆ in clinical isolates was conducted in three steps. (i) Construction of sgRNA expression cassette to *gal4*∆/∆ His1+ NAT1 sensitive strain. The SNR52 promoter was amplified with primers SNR52/F and SNR52/R_NAT1 from plasmid pV1093; the sgRNA scaffold was amplified with primers sgRNA/F_NAT1 and sgRNA/R from plasmid pV1093 (28). Then fusion PCR with nested primers SNR52/N and sgRNA/N was used to amplify the final sgRNA expression cassette. (ii) Cas9 cassette was transformed with *NAT1* sgRNA and *GAL4* sgRNA cassette and *gal4*∆*::r1HIS1r1* cassette to five His− isolates via the transient CRISPR-Cas9 system (30). To find NAT-sensitive transformants, we patched them on YPD + NAT plates. The NAT-sensitive transformants so identified were verified by primers GAL4 Check/F and CdHIS1 Check int/R; primers GAL4 Check/F and GAL4 Check int/R. (iii) Construction of *tye7*∆*::r3NAT1r*3 cassette. One of the two halves of the *tye7*∆*::r3NAT1r*3 cassette was amplified from pMH05 using primers NAT1 CRIME/F and TYE7 Del rNATrBamHI/R; the other half of the *tye7*∆*::r3NAT1r3* cassette was amplified from pMH06 using primers NAT1 CRIME/R and TYE7 Del rNATrXmaI/F (15). (iv) The Cas9 cassette was transformed with *TYE7* sgRNA cassette and *tye7*∆*::r3NAT1r3* cassette into five His− isolates via the transient CRISPR-Cas9 system (30). Transformants were verified by primers TYE7 Check/F and NAT1 check int/R; primers TYE7 Check/F and TYE7 Check int/R.

### *GAL4* complemented strain construction

Construction of *GAL4* complemented derivatives of *gal4*∆/∆ mutants was conducted in three steps. (i) Construction of sgRNA expression cassette. The SNR52 promoter was amplified with primers SNR52/F and SNR52/R_MDR1 from plasmid pV1093 (28); the sgRNA scaffold was amplified with primers sgRNA/F_MDR1 and sgRNA/R from plasmid pV1093 (28). Then fusion PCR with nested primers SNR52/N and sgRNA/N was used to amplify the final sgRNA expression cassette. (ii) Construction of *GAL4* promoter-ORF-terminator cassette and NAT marker cassette. *GAL4* promoter-ORF-terminator cassette was amplified from SC5314 genomic DNA using primers GAL4 5′→ MDR1 up/F and GAL4 3′→ pNAT 5′/R. NAT marker cassette was amplified from plasmid pNAT using primers pNAT adapt/F and pNAT 3′→ MDR1 adap/R (30). (iii) The Cas9 cassette was amplified from the plasmid pV1093 by primers CaCas9/F and CaCas9/R (28). The Cas9 cassette was transformed with *MDR1* sgRNA cassette, *GAL4* promoter-ORF-terminator cassette, and NAT marker cassette into the NAT sensitive *gal4*∆/∆ mutants *MDR1* locus of the five clinical isolates via the transient CRISPR-Cas9 system as described in Min et al. (30). Transformants were verified by primers MDR1 Check/F and MDR1 Check int/R; primers MDR1 Check/F and GAL4 Check int/R.

### Spotting plate assays

Strains were grown overnight in YPD and washed once with deionized water, then diluted in water to an optical density at 600 nm (OD_600_) of 3.0. Subsequently, fivefold dilutions were spotted onto the specified media using a multichannel pipette. Plates were incubated for 2 days at 30°C.

### RNA extraction

For Nanostring experiments, strains were cultured overnight in 5 mL YPD liquid medium at 30°C in a tissue culture rotator. Cultures of 25 mL of pre-warmed YPD in 125 mL flasks were inoculated to an OD_600_ of 0.2. The cultures were grown for 4 hours at 30°C with vigorous shaking at 225 rpm. For RNA-seq analysis, wild-type and mutant strains were grown overnight in 5 mL YPD liquid medium in a tissue culture rotator at 30°C. Overnight cultures were washed with distilled water and diluted to an OD_600_ of 0.2 in 25 mL of YPD medium. Cells were grown for 4 hours with vigorous shaking at 225 rpm at 30°C.

RNA extraction followed the method of Cravener et al. (31). Triplicate cultures were prepared and incubated from the same overnight cultures for each SC5314 and P87 WT, *tye7*Δ/Δ, *gal4*Δ/Δ, and *gal4*Δ/Δ *tye7*Δ/Δ strains. Cells were obtained from vacuum filtration and quickly frozen at −80°C prior to RNA extraction. RNA extraction was employed by physically disrupting cells with zirconia beads (Ambion, Fisher Scientific, Waltham). RNA was isolated using 25:24:1 phenol:chloroform:isoamyl alcohol, followed by a Qiagen RNeasy Mini Kit (Qiagen, Venlo, Netherlands) modified procedures.

### Nanostring

Nanostring analysis was performed as described by Woolford et al. (30). Gene expression was measured using the nCounter SPRINT Profiler. For our analysis, we chose 31 target genes and 4 normalization genes (*ARP3*, *CYP1*, *HDA1*, and *TKL1*). For each Nanostring assay, 30 ng of RNA was combined with the Nanostring probe mix and incubated at 65°C overnight for 18 hours. The samples were then loaded onto the cartridge in accordance with the manufacturer’s instructions and placed into the instrument for scanning and subsequent data collection.

Heat maps of Nanostring gene expression data were generated using Multi Experiment Viewer v4.9.0 (MeV) software. The colors in the heat maps represent log_2_-transformed ratios of gene expression comparisons.

### RNA sequencing

One microgram RNA per sample was used, and sequencing libraries were generated by using NEBNext UltraTMRNA Library Prep Kit for Illumina (NEB, USA). Sequence of 150 nt was determined from both ends of each cDNA fragment using the Illumina platform. Sequencing reads were aligned to the *C. albicans* reference (Assembly A21) using HISAT2. DESeq2 R package (version 1.40.2) was utilized to conduct differential expression analysis between two groups, each with three biological replicates. For conducting gene ontology (GO) term analyses, we employed clusterProfiler (v4.8.1) in R. Specifically, and we generated a GO term library utilizing FungiDB (27) (Candida albicans.Eupath.v63) through the R AnnotationForge package (32, 33). Genes were defined by an adjusted *P*-value of less than or equal to 0.05 and by a fold change on a log_2_ scale of greater than 1 or less than −1. GO categories only with a *P*-value of less than or equal to 0.05 were considered to be significant.

### Data interpretation

Interpretations and hypotheses were always guided by the comprehensive information at the Candida Genome Database (26), FungiDB (27), and the KEGG database (34). GO term enrichments were determined with the GO Termfinder tool at the Candida Genome Database.

## RESULTS

### Growth phenotypes of *tye7*Δ/Δ and *gal4*Δ/Δ mutants in diverse strains

To test genotype-phenotype variation among clinical isolates, we chose five strains used in our previous studies (15): SC5314 (clade 1, bloodstream isolate, and reference strain), P76067 (clade 2 and bloodstream isolate), P57055 (clade 3 and bloodstream isolate), P87 (clade 4 and oral isolate), and P75010 (clade 11 and bloodstream isolate). We generated homozygous *gal4*Δ/Δ, *tye7*Δ/Δ, and *gal4*Δ/Δ *tye7*Δ/Δ mutants in each background and assayed growth on several hexose sugars.

**Fig 1:**
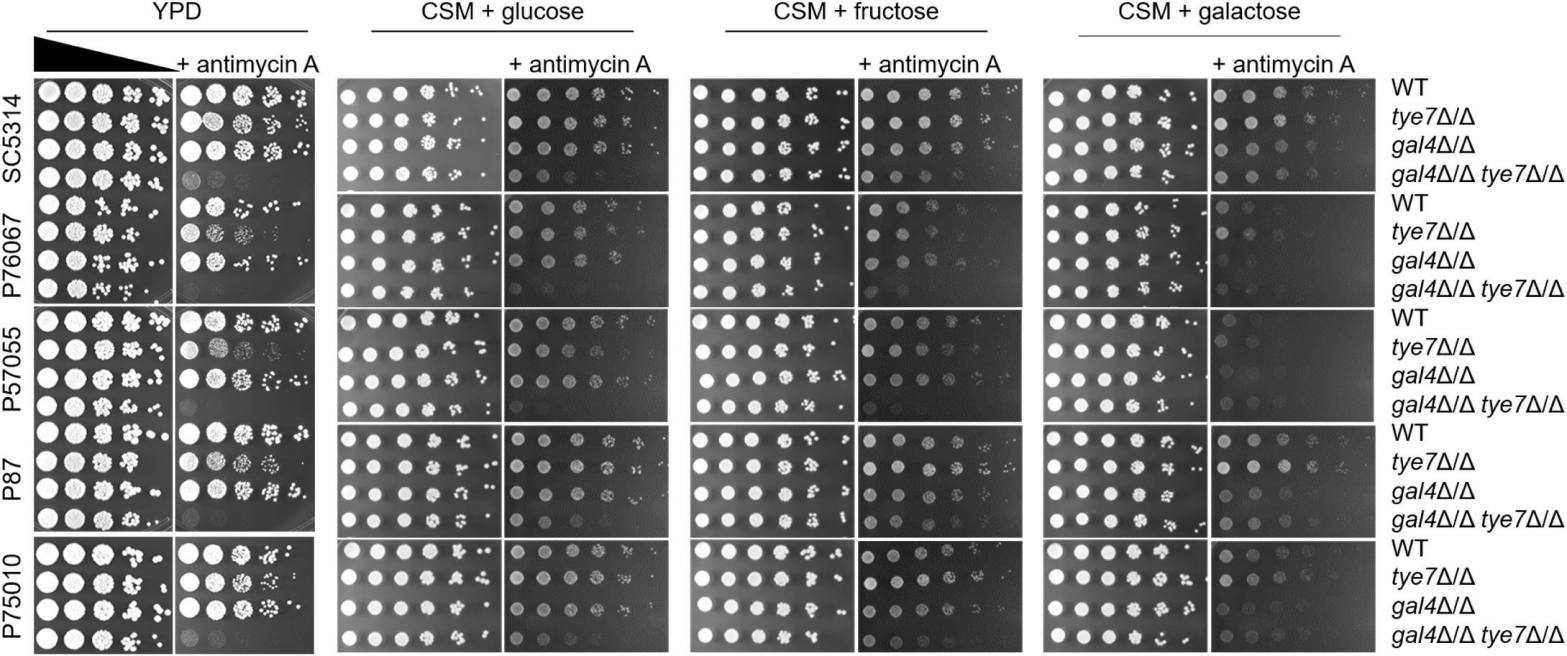
Growth impact of Tye7 and Gal4 in five strain backgrounds. Fivefold serial dilutions of the five clinical isolates and their *tye7*Δ/Δ, *gal4*Δ/Δ, and *gal4*Δ/Δ *tye7*Δ/Δ mutant derivatives were spotted on solid media with the indicated composition. Growth was visualized after 48 hours of incubation at 30°C. Media included YPD, CSM + glucose, CSM + fructose, and CSM + galactose. Pairs of plates were prepared without or with the respiration inhibitor antimycin A. Strain backgrounds are indicated on the left; relevant genotypes are indicated on the right.

All mutant strains grew comparably to their respective wild types on the rich glucose medium YPD (Fig. 1). To assay fermentative growth, we added the oxidative phosphorylation inhibitor antimycin A (5). On YPD + antimycin A, *tye7*Δ/Δ single mutants presented some variation in growth among the strains; *gal4*Δ/Δ single mutants did not; and *gal4*Δ/Δ *tye7*Δ/Δ double mutants presented severe growth defects (Fig. 1). Similar results were obtained in synthetic complete supplement mixture (CSM) medium with glucose as a carbon source (Fig. 1). These growth tests are consistent with the idea that Tye7 and Gal4 have overlapping roles in activation of glycolytic genes in multiple *C. albicans* isolates.

We also tested the growth of this strain set on fructose in a synthetic CSM medium (Fig. 1). All strains grew comparably on CSM fructose without antimycin A. On CSM fructose + antimycin A, neither *tye7*Δ/Δ nor *gal4*Δ/Δ mutants presented growth defects, and the *gal4*Δ/Δ *tye7*Δ/Δ double mutants grew more poorly than the respective single mutants or wild type (Fig. 1). These outcomes echoed the findings on glucose media in support of overlapping roles for Tye7 and Gal4 among multiple clinical isolates.

Finally, we tested the growth of this strain set on galactose in synthetic CSM medium (Fig. 1). All strains grew comparably on CSM galactose without antimycin A. On CSM galactose + antimycin A, the *tye7*Δ/Δ mutants grew as well or better than their respective wild types. Some *gal4*Δ/Δ strains presented growth defects on CSM galactose + antimycin A, as detailed below. All *gal4*Δ/Δ *tye7*Δ/Δ double mutants grew comparably to their respective *gal4*Δ/Δ single mutants on this medium. These results suggest that Gal4 may have a role related to galactose fermentation.

**Fig 2:**
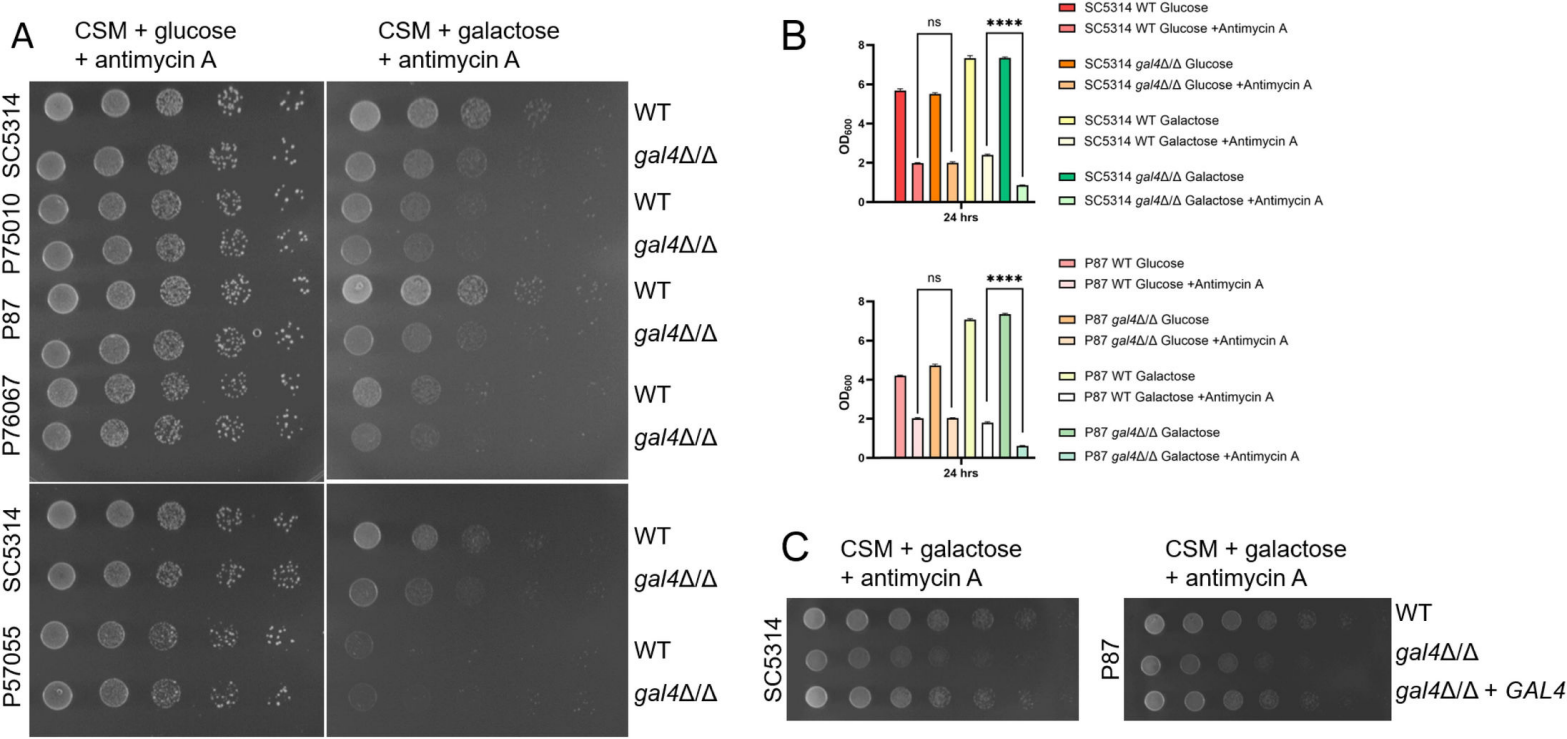
Growth impact of Gal4 on galactose. (**A**) Five-fold serial dilutions of the five clinical isolates and their *gal4*Δ/Δ mutant derivatives were tested on solid CSM + glucose + antimycin A or CSM + galactose + antimycin A, as indicated. Growth was visualized after 48 hours of incubation at 30°C. Strain backgrounds are indicated on the left; relevant genotypes are indicated on the right. (**B**) The WT and *gal4*Δ/Δ derivatives of SC5314 (upper graph) and P87 (lower graph) were grown in CSM liquid media with 0.2% glucose or 0.2% galactose, prepared with or without antimycin A, as indicated. Cultures were inoculated from a washed YPD overnight to an optical density at 600 nm (OD_600_) of 0.1, and growth was measured by OD_600_ after 24 hours at 30°C. Results of triplicate experiments are shown. Statistical analysis was conducted between SC5314 or P87 WT and *gal4*Δ/Δ strains grown in glucose or galactose media supplemented with antimycin A using an unpaired *t*-test. ns: not significant, ****, *P*-value < 0.0001. (**C**) Fivefold serial dilutions of the wild-type, *gal4*Δ/Δ mutant, and *gal4*Δ/Δ + *GAL4* complemented derivatives were tested for growth on solid CSM + galactose + antimycin A. Growth was visualized after 48 hours of incubation at 30°C. Strain backgrounds are indicated on the left; relevant genotypes are indicated on the right.

To visualize possible *gal4*Δ/Δ mutant growth defects more clearly, we plated each wild-type and derived *gal4*Δ/Δ mutant strain side by side (Fig. 2A). Growth of all wild-type and *gal4*Δ/Δ strains was comparable on CSM glucose + antimycin A. However, wild-type strains grew better than *gal4*Δ/Δ strains on CSM galactose + antimycin A for the SC5314, P75010, P87, and P76067 backgrounds. The P57055 wild-type strain grew more poorly on CSM galactose + antimycin A than the other wild types, possibly as a result of its homozygous alleles truncating C1_02500W/orf19.2939/*FMP46* and C4_01140C/orf19.4676/*MDM35* (12), both of which may affect mitochondrial function. The P57055 wild-type and *gal4*Δ/Δ strains grew equally poorly on this medium. We used two strain backgrounds, SC5314 and P87, to verify with liquid culture growth assays that each *gal4*Δ/Δ mutant grew more poorly than its respective wild type in CSM galactose + antimycin A but had no growth defect in CSM galactose (without antimycin A) or in CSM glucose + antimycin A (Fig. 2B). In these backgrounds, we also verified that introduction of a wild-type *GAL4* allele from the SC5314 background restored growth on CSM galactose + antimycin A (Fig. 2C). These results indicate that, in several strains, Gal4 promotes galactose metabolism to a greater extent than glucose metabolism.

Overall, the results of this analysis support the conclusion that, in multiple clinical isolates, Tye7 and Gal4 have overlapping positive roles in promoting fermentative growth. The results also indicate that, in several clinical isolates, Gal4 has a positive role in fermentative growth on galactose, a role not shared with Tye7.

### Gene expression impact of *tye7*Δ/Δ, *gal4*Δ/Δ, and *gal4*Δ/Δ *tye7*Δ/Δ among five strains

To profile glycolytic gene expression in glucose (YPD) medium, we conducted Nanostring analysis in *tye7*Δ/Δ, *gal4*Δ/Δ, and *gal4*Δ/Δ *tye7*Δ/Δ mutants from the five strain backgrounds. We used Nanostring probes to measure expression of 35 genes (Table S2). The gene set included 14 glycolytic genes, most of which are direct targets of both Tye7 and Gal4 (5), 17 other genes that were regulated by Tye7 or Gal4 in preliminary RNA-seq analysis, and four control genes for normalization.

**Fig 3:**
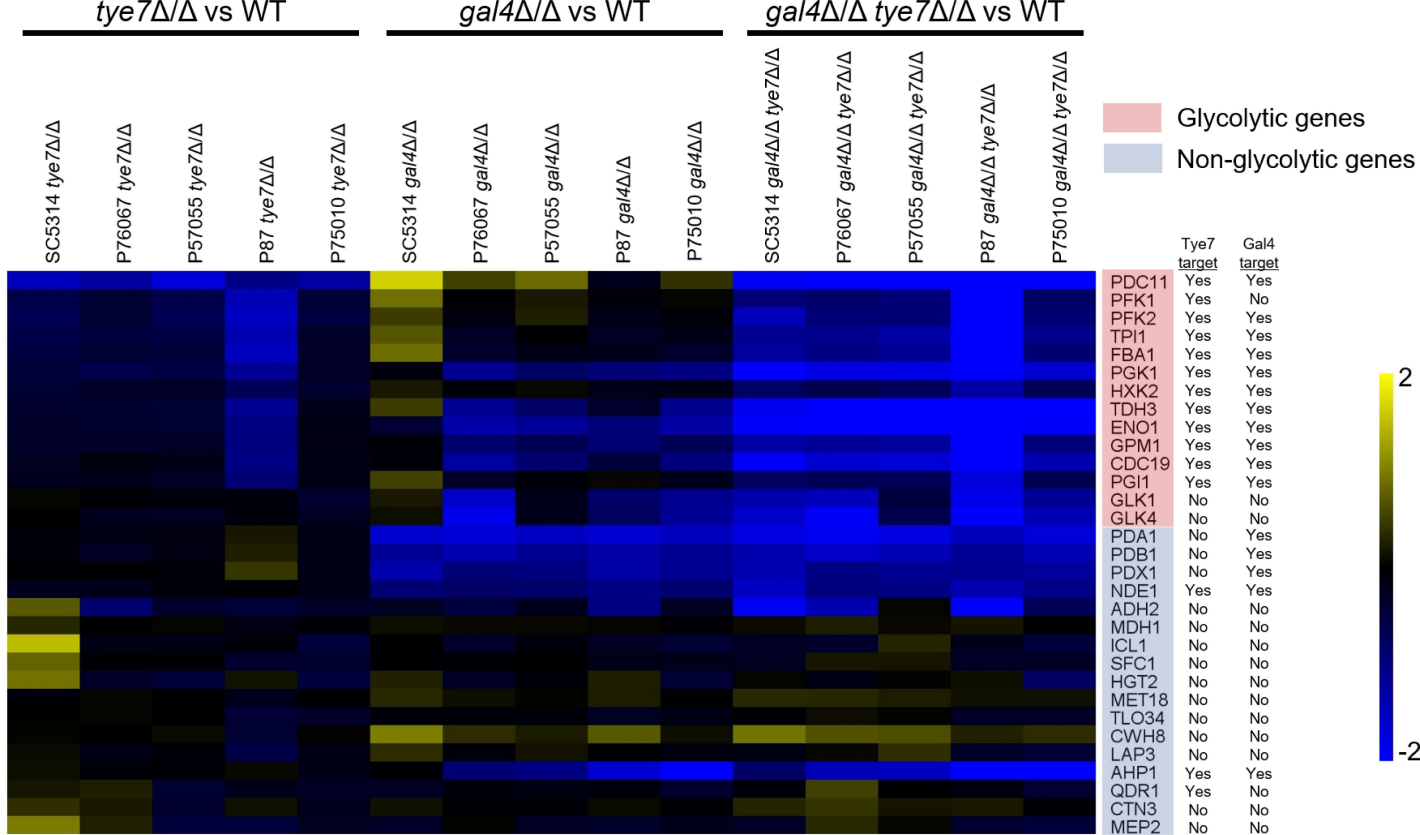
Gene expression impact of *tye7*Δ/Δ, *gal4*Δ/Δ, and *gal4*Δ/Δ *tye7*Δ/Δ mutants of clinical isolates. Log_2_-fold changes in the expression of glycolytic genes, as analyzed by Nanostring from biological triplicate RNA samples, are presented as a heat map. Strains included SC5314, P76067, P57055, P87, and P75010 clinical isolates and their *tye7*Δ/Δ, *gal4*Δ/Δ, and *gal4*Δ/Δ *tye7*Δ/Δ mutant derivatives. Cells for RNA samples were grown in YPD liquid medium at 30°C for 4 hours. Nanostring probe genes are labeled on the right with red box (glycolytic genes) or blue box (non-glycolytic genes). Probe genes that represent directly bound targets of Tye7or Gal4 are indicated on next to probe gene names based on ChIP-chip data from Askew et al. (5).

**Fig 4:**
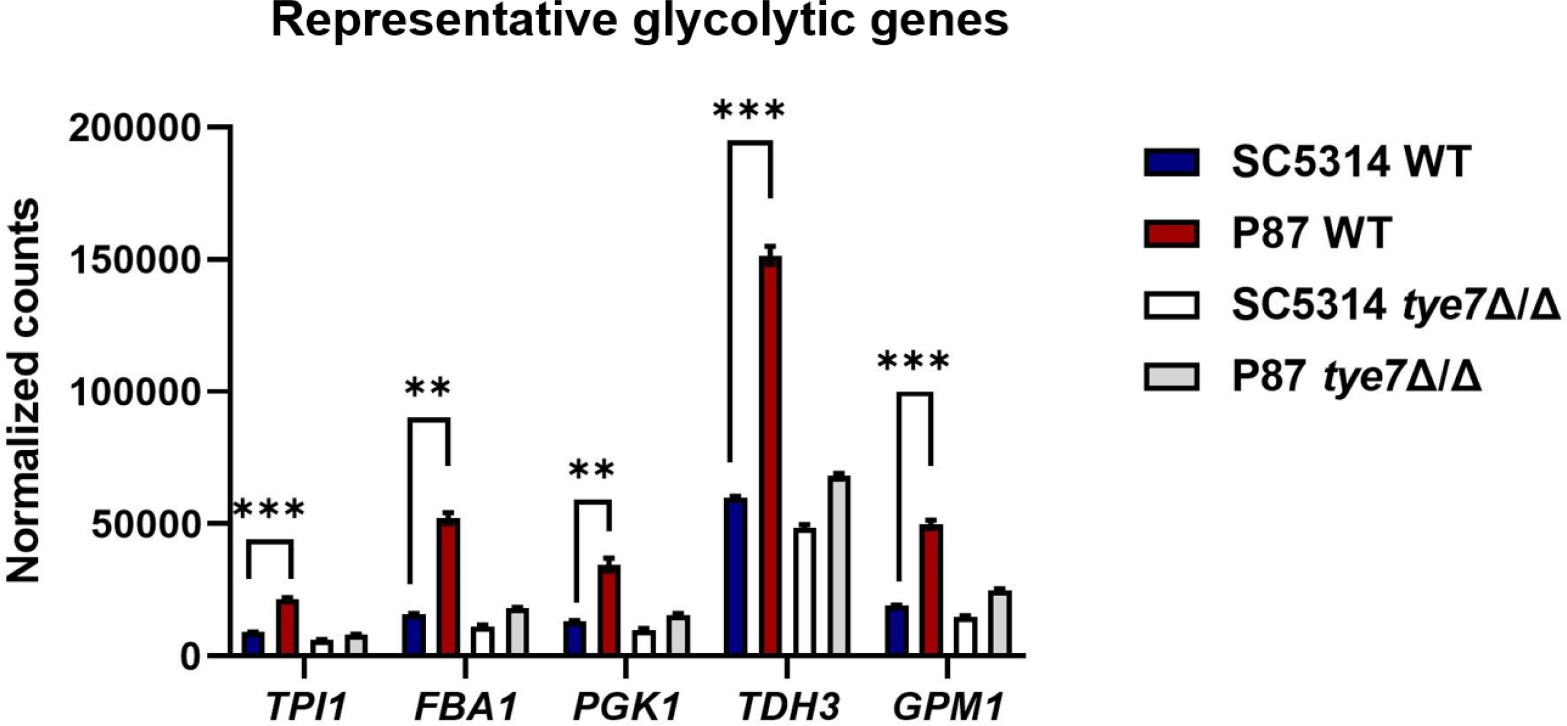
Nanostring probe counts for select glycolytic genes. Normalized counts for five representative genes as measured by Nanostring are shown. Strains included wild-type SC5314 and P87 and their *tye7*Δ/Δ derivatives. Cells were grown for triplicate RNA samples in YPD at 30°C for 4 hours. An unpaired *t*-test was performed to compare normalized counts between SC5314 and P87. Asterisks denote statistically significant differences. **, *P*-value < 0.01; ***, *P*-value < 0.001.

The *tye7*Δ/Δ mutation had little gene expression impact in most strains (Fig. 3). However, in the P87 background, the *tye7*Δ/Δ mutation caused downregulation of multiple glycolytic genes. The greater fold change in the P87 background compared to other backgrounds may reflect elevated glycolytic gene expression in the wild type or more severely diminished expression in the *tye7*Δ/Δ mutant. Probe counts for five glycolytic genes indicate that there is an elevated expression in wild-type P87 compared to wild-type SC5314 (Fig. 4). The results indicate that, under these growth conditions, the gene expression impact of a *tye7*Δ/Δ mutation is effectively buffered in most strains, though not in strain P87.

The *gal4*Δ/Δ mutation caused detectable downregulation of several glycolytic genes in the four strains P76067, P57055, P87, and P75010 (Fig. 3). The specific genes affected*—PGK1*, *TDH3*, and *ENO1*—all function in the triose portion of glycolysis and are activated by both Gal4 and Tye7 (5). This segment of the pathway leads to pyruvate dehydrogenase, specified by Gal4 direct target genes *PDA1*, *PDB1*, and *PDX1* (5). The outlier strain, in which multiple glycolytic genes were slightly upregulated in the *gal4*Δ/Δ mutant, was the reference strain SC5314. Upregulation of these genes in the SC5314 *gal4*Δ/Δ mutant may represent a compensatory increase in Tye7 activity.

The *gal4*Δ/Δ *tye7*Δ/Δ double mutants had similar gene expression impact in all strains examined (Fig. 3). Specifically, most glycolytic genes were downregulated significantly. For several genes, the fold change was greater in P87 than in the other strains. This difference reflects elevated glycolytic gene expression in wild-type P87 because normalized probe counts of all *gal4*Δ/Δ *tye7*Δ/Δ double mutants were similar (Table S2). There was little difference between SC5314 and the other strains, in keeping with the idea that glycolytic gene upregulation in SC5314 *gal4*Δ/Δ mutant was due to compensatory activity of Tye7.

We see two examples of genotype-phenotype variation in the gene expression analysis above. First, the *tye7*Δ/Δ mutation caused a larger fold change defect in strain P87 than in the other strains. Second, the *gal4*Δ/Δ mutation has a distinct gene expression impact in the reference strain SC5314. Overall, the gene expression data support the conclusion from growth phenotypes that Tye7 and Gal4 have overlapping roles in glycolytic gene expression in all five strains.

### RNA-seq analysis in two strain backgrounds

To gain a broader view of genotype-phenotype relationships, we conducted RNA-seq analysis of *tye7*Δ/Δ, *gal4*Δ/Δ, and *gal4*Δ/Δ *tye7*Δ/Δ mutants (Table S3). Cells were grown for 4 hours in glucose-based YPD medium at 30°C prior to RNA extraction. We used the SC5314 and P87 strain backgrounds, which represent extremes from the Nanostring gene expression analysis above. SC5314 was distinct in its *gal4*Δ/Δ phenotype; P87 was distinct in its *tye7*Δ/Δ phenotype.

**Fig 5:**
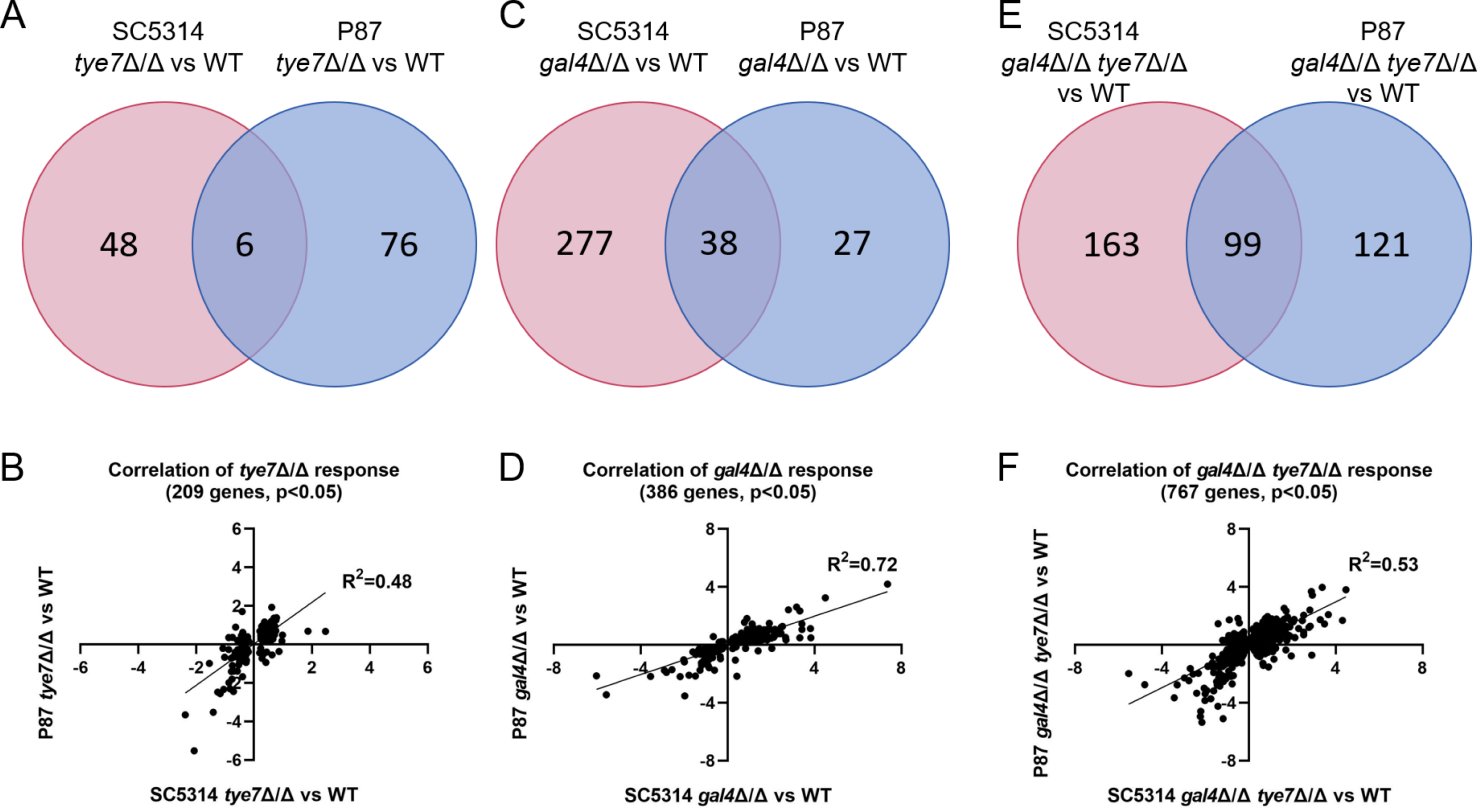
RNA-seq comparison of *tye7*Δ/Δ, *gal4*Δ/Δ, and *gal4*Δ/Δ *tye7*Δ/Δ mutants of SC5314 and P87. (**A, C, and E**) Conventional thresholds were used to define significantly up- and downregulated genes (log_2_ fold change > 1 or < −1, and adjusted *P* < 0.05) in *tye7*Δ/Δ, *gal4*Δ/Δ, and *gal4*Δ/Δ *tye7*Δ/Δ mutants compared to their respective wild types, SC5314 or P87. Venn diagrams depict the genes whose expression is affected by each mutation in both strain backgrounds. (**B, D, and F**) Regression analysis shows the number of significantly regulated (adjusted *P* < 0.05) genes, regardless of fold change, in each mutant strain. The *X* and *Y* axes represent mutant vs wild type log_2_ fold change for SC5314 and P87, respectively.

Compared to the wild type, the SC5314 *tye7*Δ/Δ mutation affected expression of 54 genes; the P87 *tye7*Δ/Δ mutation affected expression of 82 genes (Fig. 5A; log_2_ fold change > 1 or < −1; adjusted *P* < 0.05). Affected genes in SC5314 were enriched for GO terms related to carbohydrate transport and fatty acid catabolism (Table S3). Affected genes in P87 were enriched for GO terms related to purine nucleotide catabolism, capturing mainly glycolysis pathway genes (Table S3). Only six genes were affected in both strain backgrounds, including Tye7 direct target *QDR1*, and GO term enrichments were weak (Table S3). These genome-wide results are consistent with the conclusion from Nanostring analysis that *tye7*Δ/Δ impact is distinct in the SC5314 and P87 backgrounds.

The SC5314 *gal4*Δ/Δ mutation affected expression of 315 genes; the P87 *gal4*Δ/Δ mutation affected expression of 65 genes (Fig. 5C; log_2_ fold change > 1 or < −1; adjusted *P* < 0.05). Affected genes in SC5314 were enriched for GO terms related to carboxylic acid metabolism (Table S3). Affected genes in P87 were enriched for GO terms related to pyruvate and acetyl-CoA metabolism (Table S3). We found that 38 genes were affected in both strain backgrounds, including several Gal4 direct targets (*CTA26, orf19.3461, PDA1, PDB1*, and *PDX1*). GO term enrichments for this common gene set were weak (Table S3), though the gene set included pyruvate dehydrogenase genes *PDA1, PDB1*, and *PDX1*. As for the *tye7*Δ/Δ analysis above, these results are consistent with the conclusion from Nanostring analysis that *gal4*Δ/Δ impact is distinct in the SC5314 and P87 backgrounds.

The SC5314 *gal4*Δ/Δ *tye7*Δ/Δ double mutation affected expression of 262 genes; the P87 *gal4*Δ/Δ *tye7*Δ/Δ double mutation affected expression of 220 genes (Fig. 5E; log_2_ fold change > 1 or < −1; adjusted *P* < 0.05). Affected genes in SC5314 were enriched for GO terms related to carboxylic acid metabolism, representing an amalgam of glycolysis pathway genes and fatty acid catabolism genes (Table S3). Affected genes in P87 were enriched for GO terms related to pyruvate metabolism and purine nucleotide catabolism, both groups including glycolysis pathway genes (Table S3). We found that 99 genes were affected in both strain backgrounds, a greater level of consistency (26% overlap) than observed with either single mutant (5%–11%). Common upregulated genes were enriched for GO terms related to carboxylic acid metabolism and amino acid catabolism. Common downregulated genes included glycolytic genes and pyruvate metabolic genes, as observed with Nanostring assays (Fig. 3). All told, among downregulated genes, there were 15 Gal4 direct targets and 15 Tye7 direct targets. These results align with the idea that Gal4 and Tye7 have overlapping or compensatory functions (5), so the double mutant phenotype has greater consistency among strains than the single mutant phenotypes.

We used a second approach to assess the consistency of mutant gene expression impact. We compared the fold change of differentially expressed genes chosen solely for an adjusted *P*-value < 0.05 in both backgrounds, without consideration of expression fold changes. Our previous application of this approach showed that gene expression impact of *hgc1*Δ/Δ, *efg1*Δ/Δ, or *sef1*Δ/Δ mutations in different strain backgrounds correlated well (*R*^2^ ≥ 0.75), while that of an *nrg1*Δ/Δ mutation correlated poorly (*R*^2^ = 0.38) (35, 36). For the *tye7*Δ/Δ mutation, we found the correlation to be relatively weak (*R*^2^ = 0.48; Fig. 5B). For the *gal4*Δ/Δ mutation, the correlation (*R*^2^ = 0.72; Fig. 5D) was stronger. The analysis revealed that many *gal4*Δ/Δ-dependent expression changes were simply smaller for P87 than for SC5314 (Fig. 5D). These strain-independent Gal4 targets were enriched for GO terms related to carboxylic acid metabolism and amino acid catabolism (Table S3). Such genes were largely upregulated in the mutants, a response that may enable the use of amino acids in the medium for carbon. Whereas *gal4*Δ/Δ gene expression impact appeared quite different in SC5314 and P87 based on Nanostring profiles of glycolytic genes, it is overall as similar as seen for other TF mutants compared across strains (35, 36) based on RNA-seq. Finally, examination of fold change of *gal4*Δ/Δ *tye7*Δ/Δ-affected genes chosen solely for adjusted *P*-value (Fig. 5F) showed a modest correlation between the strains (*R*^2^ = 0.53), more similar to that observed above with the *tye7*Δ/Δ mutants than with the *gal4*Δ/Δ mutants. Genes affected by the double mutation in both strains included 23 of the 40 glycolysis pathway genes listed in the KEGG database (34). The consistency of the double mutant defect in hexose fermentative growth is reflected in the double mutant gene expression impact.

## DISCUSSION

Here, we have used two well-understood transcription factors, Tye7 and Gal4, to explore the impact of strain variation on central metabolic regulation. The overall relationship of Tye7 and Gal4 to glycolytic gene expression was consistent among strains. At the level of biological phenotype, a severe glucose fermentative growth defect was observed only for *tye7*Δ/Δ *gal4*Δ/Δ double mutants. At the level of gene expression, glycolytic gene RNAs were more severely reduced in *tye7*Δ/Δ *gal4*Δ/Δ double mutants than in either single mutant. These results confirm that Tye7 and Gal4 have overlapping or compensatory roles in glycolytic gene activation in multiple *C. albicans* strains.

There were two cases in which gene expression changes in single gene mutants were strain-specific. One was the P87 *tye7*Δ/Δ mutant, in which glycolytic genes were downregulated to a greater extent than in the *tye7*Δ/Δ mutants in other strains. The P87 wild type also presented higher overall levels of glycolytic gene RNAs than the other strains. A simple explanation is that the Tye7 pathway has elevated activity in P87 under our growth conditions. The comparison of RNA levels in P87 and SC5314 is consistent with this explanation. Among the 271 Tye7 direct targets identified through ChIP-chip by Askew et al. (5), we find that 64 genes are significantly upregulated, and only 13 genes are significantly downregulated in the P87 wild type compared to the SC5314 wild type (see Table S3). In addition, among the 52 genes that are significantly downregulated in the P87 *tye7*Δ/Δ mutant, we find that 26 genes are significantly upregulated, and only three genes are significantly downregulated in the P87 wild type compared to the SC5314 wild type (see Table S3). Finally, *TYE7* RNA levels are upregulated 1.8-fold in P87 compared to SC5314. These observations, though not proof, are consistent with the idea that P87 has elevated Tye7 activity compared to the other strains we examined. Perhaps the P87 lineage encountered prolonged hypoxic growth, which could select for increased Tye7 activity (5, 7, 8).

A second example of a strain-specific gene expression change in single gene mutants came from the SC5314 *gal4*Δ/Δ mutant. A few glycolytic genes (*PGK1*, *ENO1*, and *GPM1*) were slightly downregulated in *gal4*Δ/Δ mutants of most strains but not SC5314. This difference was observed in both Nanostring and RNA-seq data sets, an indication that it is not an artifact of probe function or normalization. These genes are direct targets of both Gal4 and Tye7 (5) and are downregulated similarly in the *tye7*Δ/Δ *gal4*Δ/Δ double mutants of SC5314 and the other strains. Perhaps Tye7 has a more effective *gal4*Δ/Δ-compensatory response at these promoters in SC5314 than in the other strains.

One unexpected outcome of these studies is the finding that *GAL4* is required for wild-type levels of fermentative growth on galactose in several strains. We emphasize that this result does not contradict any studies of Gal4 rewiring between *S. cerevisiae* and *C. albicans* (5, 10) or does the result indicate that Gal4 controls the classic targets *GAL1*, *GAL7*, and *GAL10* in any *C. albicans* strain. A simple possibility is that glycolytic genes are more dependent on Gal4 than Tye7 during growth of most strains on galactose, as we found during growth on glucose. In addition, poor galactose uptake by *C. albicans*, as suggested by Askew et al. (5), may magnify the impact of reduced glycolytic gene expression on galactose fermentation.

Our results here extend the genotype-phenotype puzzle that has emerged from multi-strain gene regulatory analysis: the gene expression impact of a deletion mutant varies extensively among *C. albicans* genetic backgrounds. This puzzle was evident in pioneering studies from Morschhauser and Rogers on Mrr1-regulated genes (37), and in our own studies on biofilm/hyphal regulators, and iron regulators (15–17, 19, 35, 36, 38). The variation in gene expression impact can be useful, in that genes whose expression is affected by a mutation in multiple strain backgrounds can support the discovery of novel functional relationships (17, 36, 38). We think about regulatory variation as being analogous to genome content variation, notably among bacteria (39). A bacterial species’ pangenome, which is all of the genes present in any member of the species, includes core genes (carried by all members) and accessory genes (carried by some members and not others). Therefore, for many bacteria, the regulatory impact of a mutation may present strain-specific features because of strain-specific gene content. For *C. albicans*, we find that the regulatory impact of mutations also presents strain-specific features, though gene content is quite similar (40). Relatively small changes in the expression of network regulators can change the scope of impact of a *C. albicans* TF mutation (16). Carbon sources vary considerably among colonization sites (4), making it perhaps less surprising that central carbon regulation would present the variation we have reported here.

## Data Availability

RNA-Seq data are available through the NCBI under BioProject ID PRJNA1078822.

## SUPPLEMENTAL MATERIAL

Table S1 msphere.00579-24-s0001.xlsx Strains, oligos, and plasmids.

Table S2 msphere.00579-24-s0002.xlsx Nanostring data.

Table S3 msphere.00579-24-s0003.xlsx RNA-Seq data.

ASM does not own the copyrights to Supplemental Material that may be linked to, or accessed through, an article. The authors have granted ASM a non-exclusive, world-wide license to publish the Supplemental Material files. Please contact the corresponding author directly for reuse.
